# A deep-learning method using computed tomography scout images for estimating patient body weight

**DOI:** 10.1038/s41598-021-95170-9

**Published:** 2021-08-02

**Authors:** Shota Ichikawa, Misaki Hamada, Hiroyuki Sugimori

**Affiliations:** 1 Graduate School of Health Sciences, Hokkaido University, Kita-12, Nishi-5, Kita-ku, Sapporo, 060-0812 Japan; 2 Department of Radiological Technology, Kurashiki Central Hospital, 1-1-1 Miwa, Kurashiki, Okayama 710-8602 Japan; 3 Faculty of Health Sciences, Hokkaido University, Kita-12, Nishi-5, Kita-ku, Sapporo, 060-0812 Japan

**Keywords:** Medical imaging, Computational science

## Abstract

Body weight is an indispensable parameter for determination of contrast medium dose, appropriate drug dosing, or management of radiation dose. However, we cannot always determine the accurate patient body weight at the time of computed tomography (CT) scanning, especially in emergency care. Time-efficient methods to estimate body weight with high accuracy before diagnostic CT scans currently do not exist. In this study, on the basis of 1831 chest and 519 abdominal CT scout images with the corresponding body weights, we developed and evaluated deep-learning models capable of automatically predicting body weight from CT scout images. In the model performance assessment, there were strong correlations between the actual and predicted body weights in both chest (*ρ* = 0.947, *p* < 0.001) and abdominal datasets (*ρ* = 0.869, *p* < 0.001). The mean absolute errors were 2.75 kg and 4.77 kg for the chest and abdominal datasets, respectively. Our proposed method with deep learning is useful for estimating body weights from CT scout images with clinically acceptable accuracy and potentially could be useful for determining the contrast medium dose and CT dose management in adult patients with unknown body weight.

## Introduction

Body weight is an important parameter in the field of radiology as the dose of contrast medium^1^, management of radiation dose^2, 3^, or selection of reduced tube voltage^4^ is strongly related to body weight. However, we cannot always determine the accurate body weight at the time of computed tomography (CT) scanning, especially in emergency care. Patients in emergency care are often unresponsive and unable to state their body weight. Additionally, body weight measurement using a calibrated floor scale is limited in emergency settings. Visual estimation of body weight by medical staff is unreliable^5–7^, even though it is the most accessible method. A bedside method^8^ with anthropometric measurements to estimate body weight was reported; however, this method yields moderate accuracy and may be time-consuming.

A more accurate estimation of body weight that uses CT scan data has been shown in previous studies^9, 10^. These methods might be useful in the management of radiation dose or accurate drug dosing; however, their use in the determination of the contrast medium dose is limited as these methods require diagnostic CT scan data. Contrast-enhanced CT is frequently performed without pre-acquisition of non-contrast CT images. Time-efficient methods to estimate body weight with high accuracy before diagnostic CT currently do not exist.

In a typical CT examination, the patient is initially scanned with a “scout” or “localizer” acquisition, which is a 2D planner image acquired before diagnostic CT. If body weight can be predicted from CT scout images, radiologists or technologists could determine the appropriate dose of contrast medium before diagnostic CT. Recently, deep learning has shown many encouraging results in the field of medical image processing and analysis. Particularly, a convolutional neural network (CNN) has been shown to be well suited for object detection^11, 12^, semantic segmentation^13, 14^, image classification^15, 16^, and prediction^17, 18^ tasks in radiological research. This study aimed to develop a CNN-based method using chest and abdominal CT scout images for predicting body weight and to evaluate the correlation between actual and predicted body weights.

There are two main contributions from this study. First, we propose a new time-efficient CNN-based approach for predicting body weight with clinically acceptable accuracy. Second, we demonstrate that body weight can be estimated from CT scout images even without diagnostic scan data.

The rest of this paper is arranged as follows. We briefly review related studies in Sect. 2, the details of our CNN-based method and its evaluation are provided in Sect. 3, experimental results are described in Sect. 4, and we discuss and conclude our paper in Sects. 5 and 6, respectively.

## Related studies

Body weight estimation has attracted a lot of attention for a long time in emergency care. Here we discuss the publications that are relevant to our work and briefly compare these publications with our work.

Buckley et al. proposed equations for estimating actual body weight by measuring the abdominal circumference and thigh circumference^8^. Unfortunately, their method only showed moderate accuracy, with deviations >  ± 10 kg in 15% of male patients and 27% of female patients. Furthermore, anthropometric measurements are a burden to medical staff and time-consuming. A few publications have presented more accurate equations for estimating body weight by using diagnostic CT scan data. For instance, Geraghty et al. generated the predictive equation for men that included age, L1 cross-sectional area, spinal canal area, subcutaneous fat area, and the transverse body diameter, whereas the equation for women included only age, body circumference, and the anteroposterior diameter of L1^9^. However, manual measurements of the parameters are technically challenging and time-consuming. Gascho et al. developed a linear regression equation based on effective mAs from CT dose modulation on whole-body scans^10^. However, as they mentioned, their approach is limited to polytrauma patients who undergo whole-body CT scans and experiences variances in dose modulation strategies between vendors. Furthermore, these two methods require diagnostic CT data, and their use for determining the dose of contrast medium is limited.

In our work, we used CT scout images as input for the deep-learning approach because it was certainly acquired at the time of the CT scan, and no time-consuming measurement is required for body-weight estimation.

## Materials and methods

This retrospective study was approved by the institutional review board of Kurashiki Central Hospital, which granted permission to use pre-acquired anonymized data, and the requirement for individual informed consent was waived. All methods were carried out by following the principles of the Declaration of Helsinki.

### Subjects and CT acquisition

We retrospectively reviewed all patients who underwent chest CT between June 2019 to October 2020 (*n* = 1868) and abdominal CT between June 2019 to December 2020 (*n* = 526) for medical checkup. The inclusion criterion were accurate height and weight measured by using a calibrated scale on the same day. Thirty-seven subjects with chest datasets and seven subjects with abdominal datasets were excluded due to missing data on height and weight measured on the same day. Consideration was not taken for metallic prostheses or implantable devices. Thus, for chest datasets, the final study population consisted of 1831 (1330 men, 501 women) subjects who underwent chest CT for lung cancer screening with ages ranging from 24 to 92 years. For abdominal datasets, the final study population consisted of 519 (291 men, 228 women) subjects who underwent abdominal CT for visceral fat area measurement with ages ranging from 25 to 92 years.

CT images were acquired with an 80-detector row CT scanner (Aquilion Prime; Canon Medical Systems, Otawara, Japan). The frontal scout view of the chest and abdomen was obtained by using 120 kVp and 10 mA.

### Datasets and preprocessing

**Figure 1:**
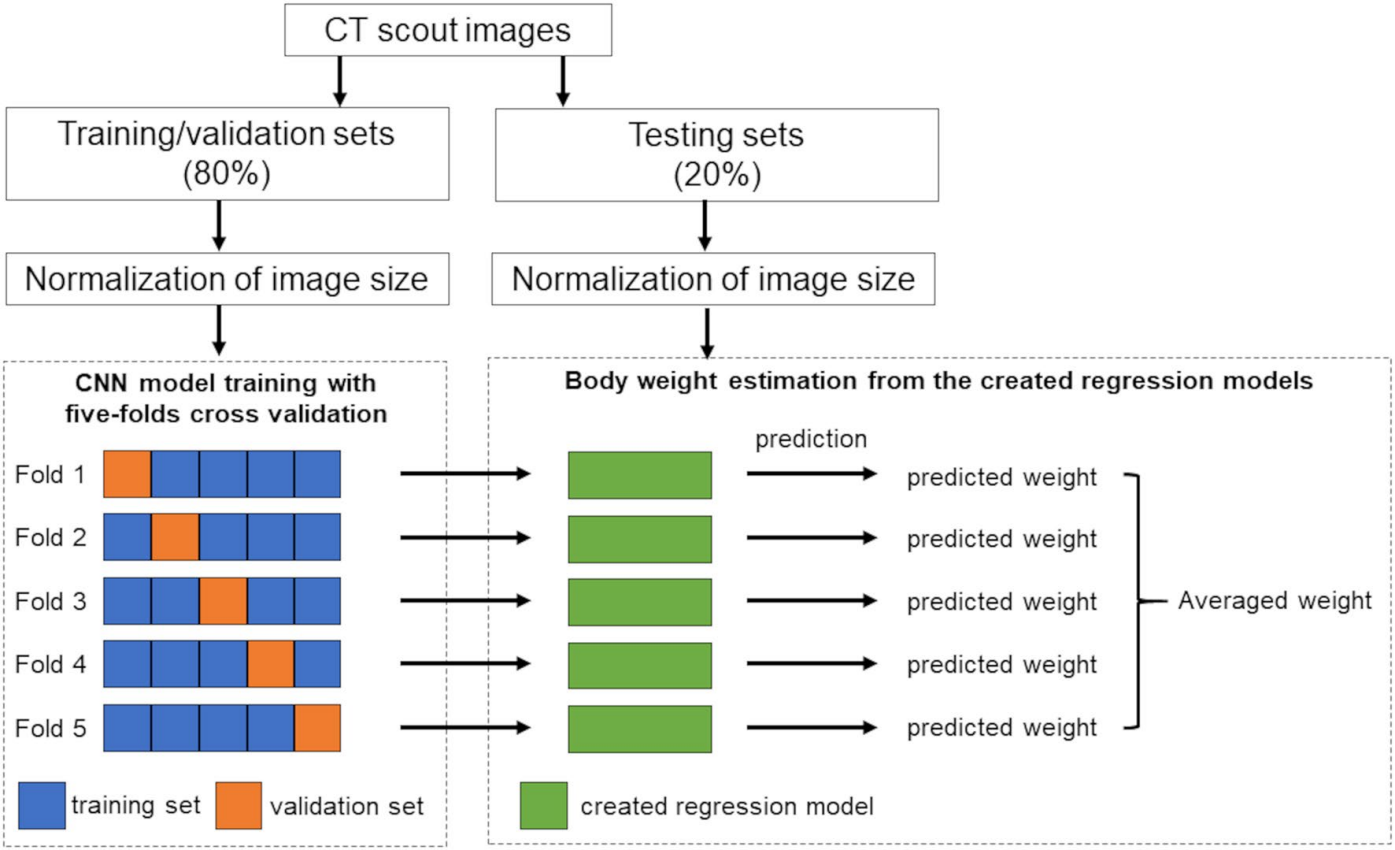
Block diagram of a convolutional neural network-based method for body-weight estimation.

Figure 1 shows a block diagram of our CNN-based method for body-weight estimation. In this study, supervised training of a CNN was performed using chest and abdominal CT scout images as input data and the corresponding body weights as reference data. The performance of our models was evaluated with other datasets that were excluded in the training datasets.

Therefore, the constructed datasets were randomly divided into training and validation sets (80%) and testing sets (20%). Of 1831 chest scout images, 1464 images were used for training and validation, and the remaining 367 images were used for testing. Of 519 abdominal scout images, 415 images were used for training and validation, and the remaining 104 images were used for testing. The data augmentation technique was not used.

The scout images were converted from a Digital Imaging and Communications in Medicine (DICOM) format to Joint Photographic Experts Group format for use in the training. The window width and level of the DICOM images were used to preset values in the DICOM tag. The first step of the preprocessing was to normalize image size before feeding them to the CNN. All scout images had a constant width of 552 pixels, but image height considerably varied. Accordingly, we resized the image height to 552 pixels through a combination of preserving their aspect ratios and using zero-padding. Then, these images were resized to 224 × 224 pixels for a transfer learning of the CNN.

### Deep convolutional neural network structure and training

**Figure 2:**
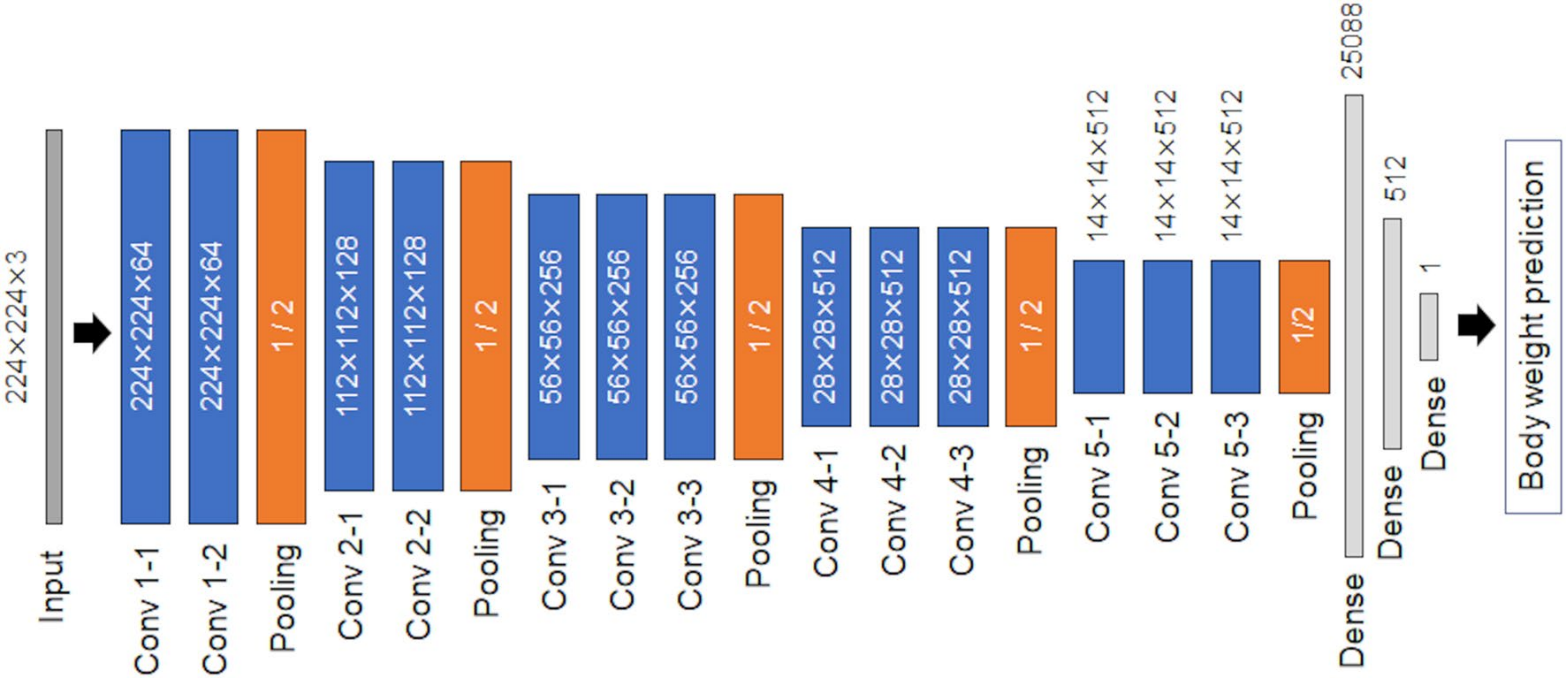
Convolutional neural network (CNN) architecture based on the VGG16 architecture. Only the fully connected layer of the original VGG16 was modified as output shape was fit to 1 for the regression model.

A regression model to predict body weight was generated based on the VGG16 architecture^19^, which was pretrained with the ImageNet database^20^. VGG16 consists of 13 convolutional layers and three fully connected layers, including rectified linear unit (ReLU) function and dropout. In this study, the fully connected layer of the original VGG16 was removed; then, the following three layers were added: (1) flatten layer, (2) fully connected dense layer with activation function “ReLU,” and (3) final fully connected dense layer with an output size of 1 (Fig. 2). Only the added fully connected layers were trained for creating the model. The loss function used was mean square error, and the Adam^21^ optimizer was used for adjusting model weights. The initial learning rate was 0.001. The learning rate was dropped by one-tenth following every three epochs of training. The maximum number of training epochs was 40, and the batch size was 32.

A *k*-fold cross-validation method with *k* = 5 was utilized for the training and validation of the CNN model. Subsequently, the model was trained five times, where four of the five sets were used for training and the remaining set was used for validation.

The CNN models were trained under the following environment: CPU, Intel Core i7-9700F; GPU, NVIDIA GeForce RTX 2070 Super 8 GB; Framework, Keras 2.3.1 with TensorFlow backend (ver.2.0.0); Language, Python 3.7.10.

### Evaluation of the created models

The created regression models were used to predict the body weights of the testing sets. The average body weight derived from the five created models was calculated for each subject. Scatter plots of the actual and predicted body weights were generated. The differences between the actual and predicted body weights were calculated. The number (%) of subjects within acceptable error was counted. According to previous reports^8^, the acceptable error was defined as 5 kg and 10 kg increments, as these seemed to be the most clinically meaningful and practical breakpoints. Then, the mean absolute error (MAE) was calculated for the chest and abdominal datasets, respectively, according to the following equation:

1 $$MAE= \frac{1}{N}\sum_{i=1}^{N}|{y}_{i}-{\widehat{y}}_{i}|$$

where N is the total number of subjects, y_i_ is the actual body weight, and ^y_i_ is the predicted body weight.

### Statistical analysis

Statistical analysis was performed using a free statistical software (R version 3.5.1, The R Foundation for Statistical Computing, Vienna, Austria). The baseline characteristics of the study subjects were expressed as the median (interquartile range) because the data were not normally distributed according to the Shapiro–Wilk test. The baseline characteristics were compared between training/validation data and testing data subsets by using the Mann–Whitney *U*-test for continuous variables. The chi-square test was used for categorical variables. The correlation between the actual and predicted body weights was evaluated by calculating Spearman’s rank correlation coefficient. The Kruskal–Wallis test was used to compare the actual body weights between the underestimated (error >  − 5 kg), acceptable (error ≤  ± 5 kg), and overestimated (error > 5 kg) cases for the actual body weight, and pairwise comparisons were made by performing the Steel–Dwass test. The level of significance was as a two-sided α ≤ 0.05.

## Results

Table 1 shows the baseline characteristics of the study subjects for chest and abdominal datasets. Of note, there are no pediatric subjects in our datasets as all subjects are aged at least 24 years for chest datasets or 25 years for abdominal datasets. This restricts the domain of our models to an adult population. The study subjects in the two subsets showed similar demographics in both chest and abdominal datasets, even though the subjects in the test abdominal datasets appeared to be slightly older (*p* = 0.019) and showed higher body mass index (*p* = 0.006).

**Table 1:** Summary of baseline characteristics of study subjects.

Region	Characteristics	Training/validation sets	Testing sets	*P*
Chest	*n*	1464	367	–
	Sex, male/female	1059/405	271/96	0.608
	Age, median (IQR) years	61 (52–69)	61 (52–69)	0.852
	Weight, median (IQR) (kg)	64.5 (57.2–72.0)	64.0 (57.2–72.1)	0.691
	Height, median (IQR) (cm)	166.8 (160.2–172.0)	166.5 (159.8–172.0)	0.514
	BMI, median (IQR) (kg/m^2^)	23.3 (21.4–25.3)	23.3 (21.4–25.6)	0.643
Abdomen	*n*	415	104	–
	Sex, male/female	232/183	59/45	0.967
	Age, median (IQR) years	57 (48–68)	60 (52–69)	0.019
	Weight, median (IQR) (kg)	63.8 (56.8–72.3)	65.3 (57.4–73.8)	0.228
	Height, median (IQR) (cm)	164.4 (155.8–171.6)	163.2 (154.5–171.3)	0.378
	BMI, median (IQR) (kg/m^2^)	23.8 (21.8–25.9)	24.9 (22.9–26.3)	0.006

*BMI*, body mass index; *IQR*, interquartile range.

**Figure 3:**
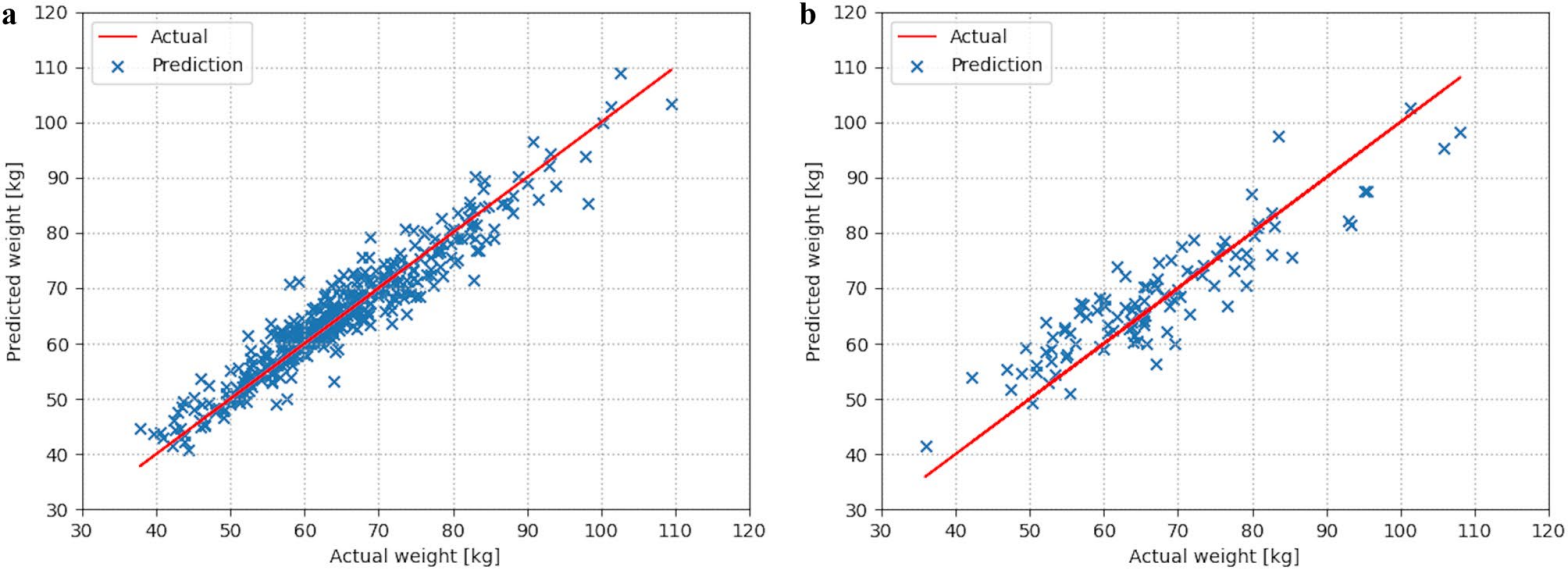
Scatterplots comparing the actual and predicted body weights in (**a**) chest and (**b**) abdominal datasets.

**Figure 4:**
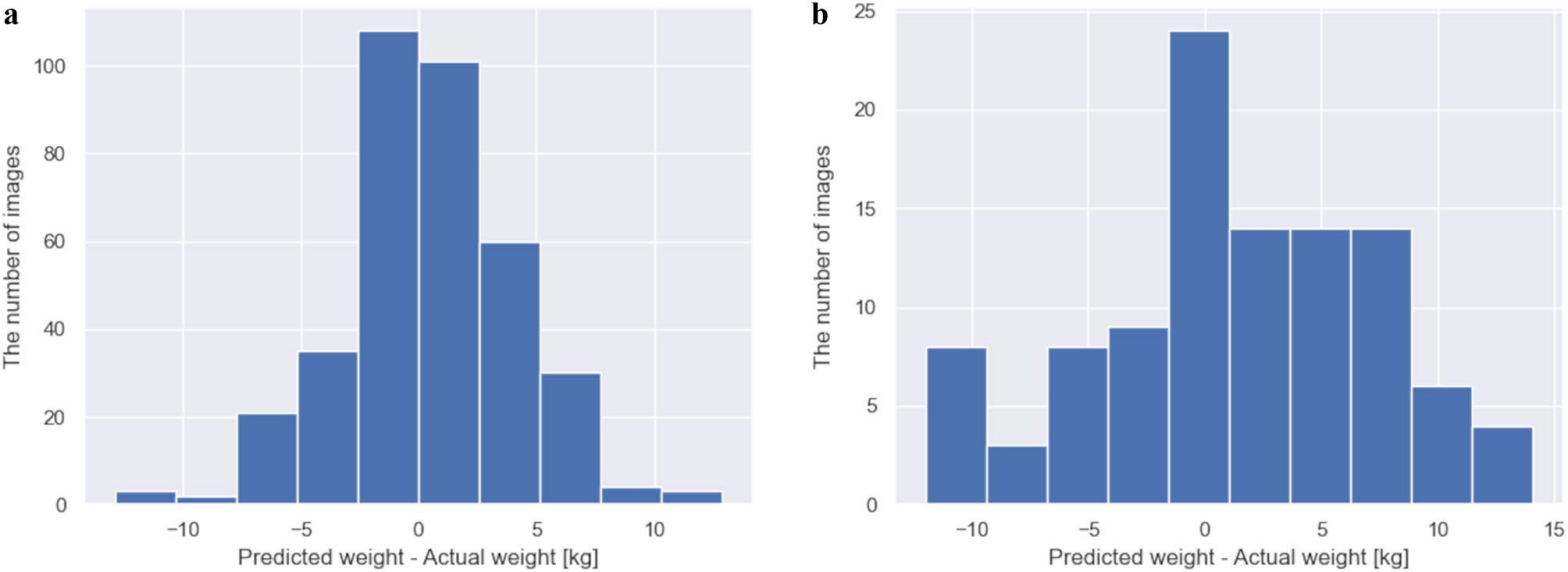
Histograms of the value of the differences between the actual and predicted body weights in (**a**) chest and (**b**) abdominal datasets.

We evaluated the created models using the testing sets for predicting body weight from CT scout images. Figure 3 shows scatterplots comparing the actual and predicted body weights in testing sets. There were strong correlations between the actual and predicted body weights (*ρ* = 0.947, *p* < 0.001 for chest; *ρ* = 0.869, *p* < 0.001 for abdomen). Figure 4 shows histograms of the value of the differences between the actual and predicted body weights. We counted the number (%) of subjects within acceptable error defined as 5 kg and 10 kg increments from actual body weight. The number (%) of subjects within ± 5 kg from the actual body weight were 302/367 (82.3%) for chest and 59/104 (56.7%) for abdominal datasets. The number (%) of subjects within ± 10 kg from the actual body weight were 361/367 (98.4%) for chest and 95/104 (91.3%) for abdominal datasets. To evaluate the performance of the regression models, we calculated the MAE for chest and abdominal datasets. The MAEs were 2.75 kg and 4.77 kg for chest and abdominal datasets, respectively.

To analyze underestimated and overestimated subjects, we compared the actual body weights between the underestimated (error > –5 kg), acceptable (error ≤  ± 5 kg), and overestimated (error > 5 kg) subjects (Tables 2 and 3). The underestimated subjects showed higher actual body weights than the acceptable subjects in both the chest and abdominal datasets (*p* < 0.001 for both), whereas the overestimated subjects showed lower actual body weights than the acceptable subjects in the abdominal datasets (*p* < 0.001). No statistically significant difference was observed between the acceptable cases and the overestimated cases in the chest datasets (*p* = 0.178).

**Table 2:** Comparisons of actual body weights between underestimated, acceptable, and overestimated cases.

Region	Group	*n*	Actual body weight, median (IQR) (kg)	*P*
Chest	Underestimated	26	76.5 (71.7–83.4)	< 0.001
	Acceptable	302	63.8 (57.0–70.8)	
	Overestimated	39	59.5 (55.0–66.5)	
Abdomen	Underestimated	16	81.1 (71.0–93.8)	< 0.001
	Acceptable	59	65.3 (61.5–72.6)	
	Overestimated	29	56.9 (52.2–61.7)	

*IQR*, interquartile range. The underestimated group was defined as the subjects with >  − 5 kg from their actual body weight. The acceptable group was defined as the subjects within ± 5 kg from their actual body weight. The overestimated group was defined as the subjects > 5 kg from their actual body weight. The *p*-values were calculated by performing the Kruskal–Wallis test.

**Table 3:** Pairwise comparisons of actual body weights between underestimated, acceptable, and overestimated cases.

Region	Pair	*t*	*p*
Chest	Underestimated versus acceptable	4.990	< 0.001
	Underestimated versus overestimated	4.533	< 0.001
	Acceptable versus overestimated	1.776	0.178
Abdomen	Underestimated versus acceptable	4.359	< 0.001
	Underestimated versus overestimated	4.767	< 0.001
	Acceptable versus overestimated	3.764	< 0.001

The underestimated group was defined as the subjects with >  − 5 kg from their actual body weight. The acceptable group was defined as the subjects within ± 5 kg from their actual body weight. The overestimated group was defined as the subjects with > 5 kg from their actual body weight. The *p*-values were calculated by performing the Steel–Dwass test.

**Figure 5:**
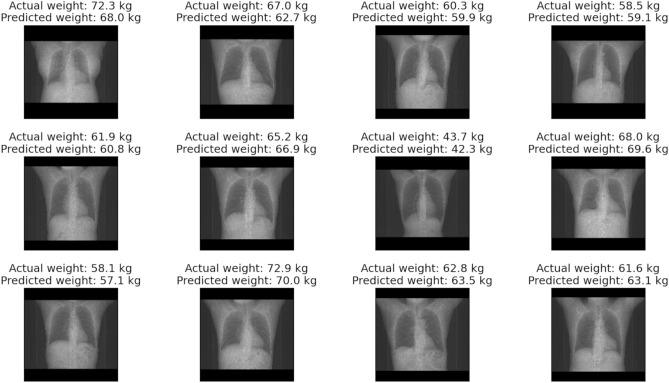
Representative cases of our CNN-based approach for predicting body weight in chest datasets. The upper row shows the actual body weight, and the lower row shows the predicted body weight from our CNN-based method.

**Figure 6:**
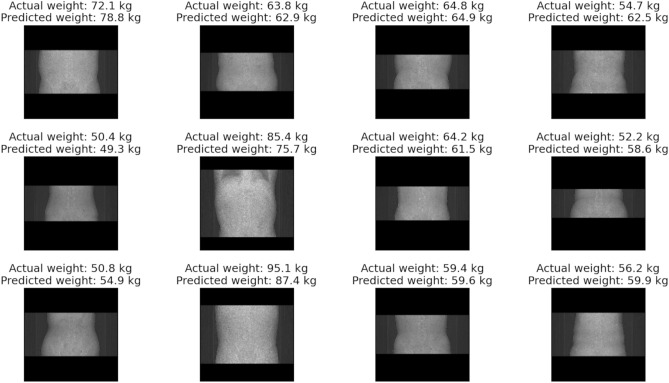
Representative cases of our CNN-based approach for predicting body weight in abdominal datasets. The upper row shows the actual body weight, and the lower row shows the predicted body weight from our CNN-based method.

Representative cases of our CNN-based approach for predicting body weight from CT scout images are shown in Figs. 5 and 6. The implementation time was very short, which was only 6.7 s for an image from image reading to providing predicted body weight.

## Discussion

Body weight is an indispensable parameter for determination of contrast medium dose, appropriate drug dosing, or management of radiation dose. In this study, the predicted body weight, by applying a deep-learning technique to chest and abdominal CT scout images, was found to be highly correlated with the actual body weight. Our models showed the MAE within 5.0 kg in both chest and abdominal datasets, even with a relatively modest training dataset size.

To the best of our knowledge, this is the first attempt to predict the body weight from CT scout images by applying a deep-learning technique. In contrast, previous studies have required diagnostic abdominal CT images^9^ or effective mAs from whole-body scan data^10^ for body-weight estimation. This indicates that our CNN-based method can predict the patient body weight, even when non-contrast CT images do not exist. In clinical radiology, we frequently perform contrast-enhanced CT immediately after the first “scout” or “localizer” acquisition without acquiring non-contrast CT images. Thus, our method could be applicable to more cases than previous proposed methods.

Fernandes et al. reported that patients’ own weight estimates are likely to be more accurate than those of physicians or nurses, if weight measurement on an accurate scale is impractical^7^. However, patients in emergency care often have difficulty in reporting their own body weight. A bedside method using supine thigh and abdominal circumference measurements by Buckley et al. yielded greater accuracy than visual body weight estimates made by physicians and nurses, but deviations >  ± 10 kg from the measured body weight were still noted in 15% of male patients and 27% of female patients^8^. An equation based on effective mAs by Gascho et al. revealed strong correlation (*r* = 0.969) between measured and predicted body weights for both women and men, with a postmortem interval of < 4 days^10^. The present study showed that deviations >  ± 10 kg from the actual body weight were noted in only 1.6% of the subjects for the chest and 8.7% for the abdomen. The correlations between the actual and predicted body weights were strong in both the chest and abdomen. These results indicate that our CNN-based method shows potential use in predicting patient body weight accurately in an adult populations with unknown bodyweights.

In this study, better correlation was observed in chest scout images than in abdominal scout images. One possible reason was that the dataset size was smaller for the abdominal scout images than for the chest scout images. Generally, the accuracy of a deep-learning neural network model is largely dependent on the size of high-quality initial training datasets^22–24^. Conversely, a previous study by Fukunaga et al. has shown a similar tendency to the present study, in which a better correlation between body weight and effective diameter was found in chest CT compared to abdominal CT^2^. Boos et al. also revealed that the effective diameter had a significantly better correlation with weight than with body mass index in chest CT scans, whereas it had a significantly better correlation with body mass index than with weight in abdominal CT scans^3^. Surprisingly, it seems that body weight should therefore be estimated not from the abdominal region but from the chest region, if the scan range includes the chest region.

To analyze underestimated and overestimated subjects, we compared actual body weights between the underestimated (error >  − 5 kg), acceptable (error ≤  ± 5 kg), and overestimated (error > 5 kg) cases. As the results, the underestimated cases showed higher actual body weights than the acceptable cases in both the chest and abdominal datasets, whereas the overestimated cases showed lower actual body weights than the acceptable cases in the abdominal datasets. These results mean that the overweight cases tended to be underestimated in both chest and abdominal datasets, whereas the underweight cases tended to be overestimated only in abdominal datasets. These phenomena would imply that there was an insufficient overweight or underweight population in the training/validation sets. Further training with large datasets, including overweight and underweight populations, should improve the model performance^22–24^.

There were several limitations in our study. First, sex was not considered in creating models to not reduce the size of training sets. Small training sets would cause decreased accuracy of the deep-learning neural network model, as mentioned above. Although an adequate number of datasets to train a medical image deep-learning system has been previously explored^22, 25^, the current study consisted of hundreds of thousands of images as many prior studies did^11–14, 17^. An equation by Gascho et al. considered sex in body weight estimation using effective mAs from CT dose modulation, according to the multivariate linear regression analysis^10^. We found that attenuation characteristics in scout images also appeared to be affected by sex due to the different body compositions, even among subjects with the same body weight. Therefore, the performance of our models could be improved by considering sex. Second, we only trained and tested our models on CT scout images of medical checkup subjects, and our results may not generalize to some clinical settings. For instance, arm raising is occasionally omitted for trauma patients in emergency care. Moreover, some patients have metallic implants in their bodies, such as the arms, heart, and spine. These variations were not included in the supervised training datasets, and therefore, the performance of our models is unclear. Finally, this was a retrospective study using training and testing sets from a single institution, and the ability of the models to generalize to CT scout images obtained at external institutions with other machines is unknown. For instance, tube current, reconstruction kernel, and window settings for scout images vary across institutions with CT scanners made by different manufactures. Generally, the performance of deep-learning models worsens when applied to previously unseen images. However, inter-vendor performance of deep learning might be enhanced via fine-tuning the model by using a small sample of the images from a different manufacturer^26^.

Even with these limitations, our study has contributed to development of a time-efficient method for predicting body weight from CT scout images. In emergency care, such a method can be used to determine the contrast medium dose for patients who are unresponsive and unable to state their body weight. In practical terms, deployment of our method in clinical use could be achieved by integrating it as an additional module on the CT scanner. As soon as a CT scout image is acquired, this module would generate the patient’s body weight for review by the radiologist or radiological technologist. The radiologists or radiological technologists could then decide to determine the contrast medium dose. Furthermore, by deploying our method as independent software, we could retrospectively obtain the patient’s body weights from previously acquired CT images. Such software could support CT dose management of individual patients. Thus, future research in this area will test the possibility of building the CNN-based method into a software application with a user-friendly interface.

## Conclusion

In this study, we developed and evaluated a CNN-based method using chest and abdominal CT scout images for predicting body weight. This method demonstrated acceptable accuracy for body-weight estimation and was highly time efficient. Our CNN-based method has the potential to be deployed in clinical settings in the future and potentially could be useful for determining the contrast medium dose and CT dose management in adult patients with unknown body weight.

## Data Availability

The code generated during the current study is available from the corresponding author on reasonable request. However, the image datasets presented in this study are not publicly available due to ethical reasons.
